# APOE genotype and Alzheimer’s immunotherapy

**DOI:** 10.18632/oncotarget.17990

**Published:** 2017-05-19

**Authors:** Joanna E. Pankiewicz, Martin J. Sadowski

**Affiliations:** Departments of Neurology, Psychiatry, and Biochemistry and Molecular Pharmacology, New York University School of Medicine, New York, NY, USA

**Keywords:** Alzheimer’s disease, amyloid related imaging abnormalities, apolipoprotein E, β-amyloid, microglia

Alzheimer's disease (AD) is a chronic neurodegenerative disease and the most common cause of dementia. AD risk is foremost modified by allelic composition of the *APOE* gene encoding apolipoprotein (apo) E-brain's main lipid carrying protein. Emerging evidence suggests that *APOE* genotype also may modulate efficacy and safety of AD immunotherapy, which is under development as a potential disease-modifying treatment.

There are three allelic forms of the *APOE* gene ε2, ε3, and ε4. AD frequency reaches 91% and 47% in *APOE* ε4 homo- and heterozygous carriers compared to 20% in non-carriers, respectively; while the age of dementia onset averages 68 and 76 years in *APOE* ε4 homo- and heterozygous carriers compared to 84 years in non-carriers, respectively [1]. A protective effect of the *APOE* ε2 allele also can be seen among *APOE* ε4 non-carriers. *APOE* genotype effecting AD predisposition results from a combination of disease specific and constitutional biological effects differentially modulated by apo E isoforms encoded by various *APOE* alleles [2]. Apo E isoforms diversely affect rate of soluble β-amyloid (Aβ) clearance from the brain interstitial space and also promote formation of Aβ plaques and vascular deposits, what effects a down-stream neurodegenerative cascade involving neurofibrillary pathology, inflammatory microglia response, and synaptic and neuronal loss. *APOE* ε4 carriers typically show much greater load of Aβ deposits compared to non-carriers. Constitutional effects of apo E implicated in AD pathogenesis concern its involvement in microglia phagocytic function, synaptic plasticity and neuronal network repair. *APOE* ε4 carriers have an attenuated reparative response to the neurodegenerative cascade induced by Aβ accumulation they are more prone to develop.

Development of anti-Aβ immunotherapy as a disease-modifying treatment is being actively pursued. One tested approach concerns intravenous administration of monoclonal antibodies (mAbs) recognizing antigens exposed in brain deposited Aβ. A modest fraction of such mAbs permeates the blood-brain barrier and upon binding Aβ plaques facilitates their clearance by macrophage transformed microglia. Bapinezumab was the first humanized mAb having this modus operandi, which came to clinical development. Its phase 2 trial evidenced potential effect of *APOE* genotype on efficacy and safety. Significant treatment effects on cognitive and functional endpoints were found in *APOE* ε4 non-carriers but not in *APOE* ε4 carriers [3], while amyloid related imaging abnormalities (ARIA) (including vasogenic edema [ARIA-E] and microhemorrhages [ARIA-H]) occurred seven and three times more frequently in *APOE* ε4 homo- and heterozygous carriers compared to non-carriers, respectively [4]. Exact pathomechanism of ARIA remains elusive but it is linked to immune response against perivascular Aβ causing transient increase in vascular wall permeability. Roughly 20% of ARIA-E affected patients reported clinical signs and symptoms. Enrolment to subsequent phase III trials was prospectively segregated based on *APOE* ε4 carrier status and the maximal bapinezumab dose was reduced from 2 mg/kg tested in phase 2 trial to 0.5 mg/kg and 1 mg/kg in *APOE* ε4 carrier and non-carrier groups, respectively [5]. Although dose reduction was a rationale measure to manage ARIA risk, in retrospect it can be viewed as one of several reasons these trials failed to meet efficacy endpoints.

Aducanumab is a newer anti-Aβ mAb, which like bapinezumab binds deposited Aβ exerting effector microglia response but can be tolerated in significantly higher doses [6]. Its phase 1b clinical trial showed dose dependent reduction of Aβ deposits. The maximal tested dose was 10 mg/kg administered every four weeks, while bapinezumab was infused quarterly. Unlike previous AD clinical trials, aducanumab development focuses on prodromal AD patients in attempt to contain Aβ pathology early and attenuate down-stream neurodegenerative cascade. In aforementioned phase 1b trial, 10 mg/kg aducanumab dose showed strong effect on cognitive endpoints. ARIA remains main adverse effect of aducanumab and its risk is mediated by the *APOE* ε4 allele. Thus, for two enrolling phase 3 trials patients are prospectively segregated based on *APOE* ε4 status. *APOE* ε4 carriers are randomized to either placebo, 3 or 6 mg/kg aducanumab doses while non-carriers to either placebo, 6 or 10 mg/kg doses.

**Figure 1:**
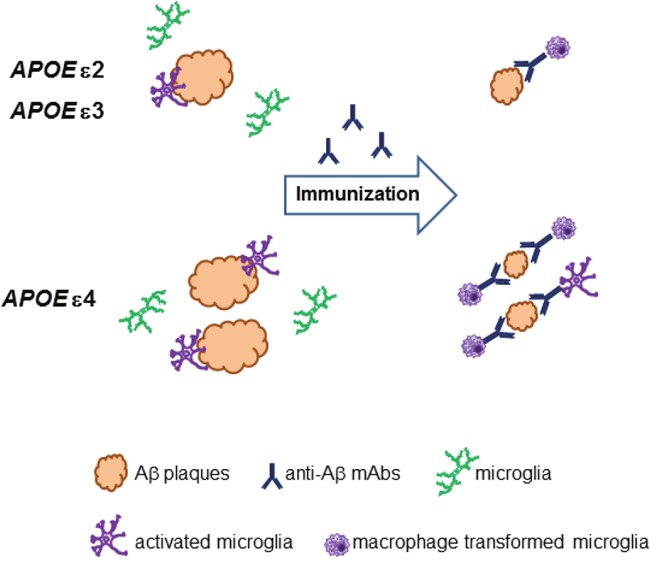
Interaction between *APOE* genotype, Aβ deposition, and microglia The *APOE* ε4 allele is associated with greater β-amyloid (Aβ) deposition than *APOE* ε2 and ε3 alleles. Aβ parenchymal plaques attract microglia cells and cause their peri-plaque activation, which is proportional to Aβ plaque load across all *APOE* genotypes. Therapeutic anti-Aβ monoclonal antibodies (mAbs) bind deposited Aβ and activate microglia cells to clear Aβ through Fc receptor-mediated phagocytosis. The *APOE* ε4 allele is associated with greater microglia activation than *APOE* alleles ε2 and ε3 resulting in greater clearance of Aβ plaque load during Aβ-directed passive immunization.

Given emerging evidence concerning effects of *APOE* genotype on efficacy and safety of AD immunotherapy we took “bedside-to-bench” approach re-testing outcomes of passive immunization in APP_SWE_/PS1_dE9_ AD transgenic mice made homozygous for each of human *APOE* alleles. We used 10D5 anti-Aβ mAb with similar modus operandi to that of bapinezumab or aducanumab. The same 10D5 mAb dose (10 mg/kg/week) was used across all *APOE* genotypes. *APOE* ε4 mice showed the greatest reduction in Aβ deposits and the most robust microglia activation adjusted for Aβ plaque load, which for the first time evidenced that the *APOE* ε4 allele mediates stronger microglia response to anti-Aβ immunotherapy and enhances microglia phagocytic effect [7] (Figure 1). As aducanumab phase III clinical trials employ Aβ PET imaging, it would be interesting to see whether reduction in Aβ load with comparable dose is higher in *APOE* ε4 carriers than non-carrier, counteracting typically higher Aβ plaque load of the former.

We also investigated effects of *APOE* genotype on vascular complications of anti-Aβ immunotherapy. Using μMRI we showed occurrence of new microhemorrhages (ARIA-H) in *APOE* ε4 mice undergoing 10D5 mAb treatment but we found no evidence of “vasogenic edema” (ARIA-E) [8]. Though ARIA-E remains the most troublesome and dose-limiting adverse effect of anti-Aβ immunotherapy its nature remains obscure largely due to absence of animal models allowing to dissect its pathogenesis. Postmortem analysis of perivascular microhemorrhages across *APOE* genotypes showed their greatest incidence in *APOE* ε2 mice evidencing for the first time the *APOE* ε2 allele as a risk factor for Aβ immunotherapy related microhemorrhages [7]. When translating these observations from transgenic mice back to humans one needs to be mindful that ε2/ε2 genotype is rare among AD patients, nevertheless careful monitoring of *APOE* ε2 carriers during clinical trials of anti-Aβ immunotherapy may effect a reduction in ARIA-H events.
